# Bis[2,4-dichloro-6-(ethyl­imino­meth­yl)phenolato-κ^2^ *N*,*O*]nickel(II)

**DOI:** 10.1107/S160053681104325X

**Published:** 2011-10-29

**Authors:** Qiu Ping Huang, Shu Hua Zhang, Jing Jing Guo, Chao Feng, Fu Shun Tang

**Affiliations:** a Key Laboratory of Non-ferrous Metal Materials and Processing Technology, Ministry of Education, College of Chemistry and Bioengineering, Guilin University of Technology, Guilin 541004, People’s Republic of China

## Abstract

In the title compound, [Ni(C_9_H_8_Cl_2_NO)_2_], the Ni^II^ ion lies on an inversion centre and is coordinated in a slightly distorted square-planar geometry by an N and an O atom from two symmetry-related bidentate 2,4-dichloro-6-(ethyl­imino­meth­yl)phenolate ligands. In the crystal structure, there are short Cl⋯Cl distances of 3.506 (1) and 3.350 (1) Å.

## Related literature

For halogen–halogen inter­actions in supra­molecular chemistry and crystal engineering, see: Cohen *et al.* (1964 ▶); Desiraju (1989 ▶); Xiao & Zhang (2008 ▶); Aakeröy *et al.* (2011 ▶).
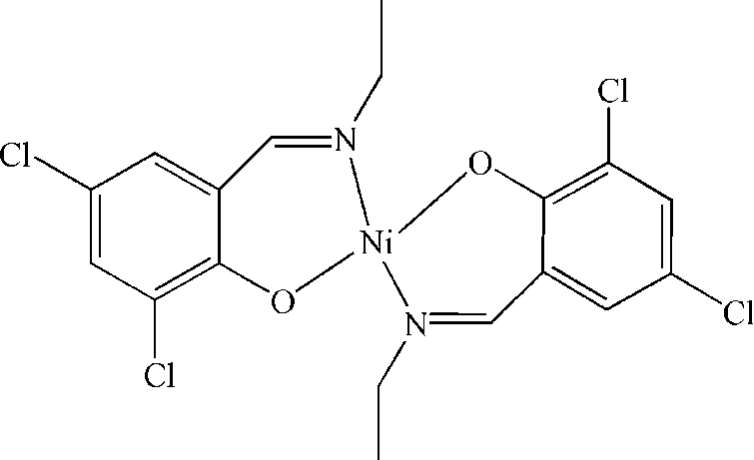


## Experimental

### 

#### Crystal data

[Ni(C_9_H_8_Cl_2_NO)_2_]
*M* *_r_* = 492.84
Monoclinic, 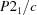

*a* = 7.5004 (6) Å
*b* = 9.3155 (7) Å
*c* = 14.1498 (12) Å
β = 103.841 (1)°
*V* = 959.94 (13) Å^3^
*Z* = 2
Mo *K*α radiation
μ = 1.58 mm^−1^
*T* = 293 K
0.32 × 0.28 × 0.26 mm

#### Data collection

Bruker SMART CCD diffractometer
Absorption correction: multi-scan (*SADABS*; Bruker, 2004 ▶) *T*_min_ = 0.612, *T*_max_ = 0.667
4890 measured reflections
1685 independent reflections
1267 reflections with *I* > 2σ(*I*)
*R*_int_ = 0.060

#### Refinement

*R*[*F* ^2^ > 2σ(*F* ^2^)] = 0.031
*wR*(*F* ^2^) = 0.055
*S* = 0.97
1685 reflections
124 parameters
H-atom parameters constrained
Δρ_max_ = 0.28 e Å^−3^
Δρ_min_ = −0.35 e Å^−3^

### 

Data collection: *SMART* (Bruker, 2004 ▶); cell refinement: *SAINT* (Bruker, 2004 ▶); data reduction: *SAINT*; program(s) used to solve structure: *SHELXS97* (Sheldrick, 2008 ▶); program(s) used to refine structure: *SHELXL97* (Sheldrick, 2008 ▶); molecular graphics: *SHELXTL* (Sheldrick, 2008 ▶); software used to prepare material for publication: *SHELXTL*.

## Supplementary Material

Crystal structure: contains datablock(s) I, global. DOI: 10.1107/S160053681104325X/lh5353sup1.cif

Structure factors: contains datablock(s) I. DOI: 10.1107/S160053681104325X/lh5353Isup2.hkl

Additional supplementary materials:  crystallographic information; 3D view; checkCIF report

## Figures and Tables

**Fig. 1.**
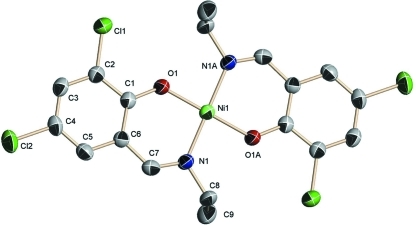
The molecular structure of (I), showing 30% probability displacement ellipsoids. H atoms are omitted.

**Fig. 2.**
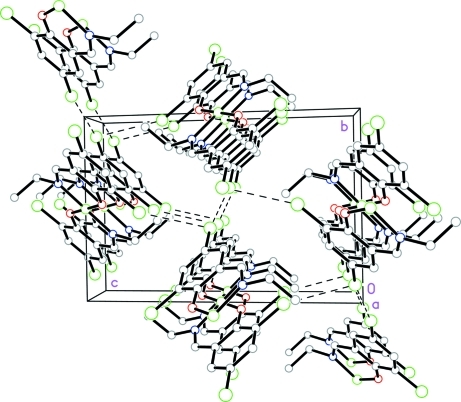
Part of the crystal structure showing short Cl···Cl contacts as dashed lines.

## supplementary crystallographic information

### Comment

Halogens have a ubiquitous presence in both inorganic and organic chemistry. Schiff bases of chloro substituents on aromatic systems have aroused interest in recent years because these halogenated compounds are an attractive target for use in supramolecular chemistry and crystal engineering wherein the halogen atoms are directly involved in forming intermolecular interactions (Cohen *et al.*, 1964; Desiraju, 1989; Xiao & Zhang, 2008; Aakeröy *et al.* 2011). The title compound, (I), contains a deprotonated 2,4-dichloro-2-ethyliminomethyl-phenol ligand, with two Cl atoms accesible for Cl···Cl interactions.

In (I), the Ni^II^ ion lies on an inversion center and is coordinated by two O and two N atoms from two symmetry related bidentate 2,4-diChloro-*N*-ethylsalicylaldimino ligands, forming a slightly distorted square-planar geometry (Fig. 1). In the crystal, there are short Cl···Cl contacts (Cl1···Cl2^i^ 3.506 (1) Å, Cl2···Cl2^ii^ 3.350 (1) Å symmetry code:(i) 1 - *x*, 1/2 + *y*, 1/2 - *z*, (ii) -*x*, -*y*, -*z*) (Fig. 2).

### Experimental

A solution of (0.191 g, 1.0 mmol) 3,5-dichloro-2-hydroxy-benzaldehyde and (0.044 g, 1 mmol) ethylamine and (0.040 g, 1 mmol) sodium hydroxide in 20 ml absolute methanol was added slowly a solution of nickel nitrate hexahydrate (0.145 g, 0.5 mmol) in methanol. The mixture was stirred for 3 h at room temperature to give a green solution which was filtered and the filtrate was left to stand at room temperature. Green block-shaped crystals suitable for X-ray diffraction were obtained by slow evaporation. yield: 78.2% (Based on Nickel). Elemental analysis calculated: C 43.83, H 3.75, N 5.68%; Found: C 43.79, H,3.78, N 5.71%.

### Refinement

H atoms were positioned geometrically and refined with a riding model, with C—H distances = 0.93–0.97 Å and with *U*_iso_(H) = 1.2*U*_eq_(C) or *U*_iso_(H) = 1.5*U*_eq_(C_methyl_).

### Crystal data

**Table:**

[Ni(C_9_H_8_Cl_2_NO)_2_]	*F*(000) = 500
*M**_r_* = 492.84	*D*_x_ = 1.705 Mg m^−^^3^
Monoclinic, *P*2_1_/*c*	Mo *K*α radiation, λ = 0.71073 Å
Hall symbol: -P 2ybc	Cell parameters from 1685 reflections
*a* = 7.5004 (6) Å	θ = 2.6–25.0°
*b* = 9.3155 (7) Å	µ = 1.58 mm^−^^1^
*c* = 14.1498 (12) Å	*T* = 293 K
β = 103.841 (1)°	Block, green
*V* = 959.94 (13) Å^3^	0.32 × 0.28 × 0.26 mm
*Z* = 2	

### Data collection

**Table:**

Bruker SMART CCD diffractometer	1685 independent reflections
Radiation source: fine-focus sealed tube	1267 reflections with *I* > 2σ(*I*)
graphite	*R*_int_ = 0.060
φ and ω scans	θ_max_ = 25.0°, θ_min_ = 2.6°
Absorption correction: multi-scan (*SADABS*; Bruker, 2004)	*h* = −8→7
*T*_min_ = 0.612, *T*_max_ = 0.667	*k* = −11→8
4890 measured reflections	*l* = −16→16

### Refinement

**Table:**

Refinement on *F*^2^	Primary atom site location: structure-invariant direct methods
Least-squares matrix: full	Secondary atom site location: difference Fourier map
*R*[*F*^2^ > 2σ(*F*^2^)] = 0.031	Hydrogen site location: inferred from neighbouring sites
*wR*(*F*^2^) = 0.055	H-atom parameters constrained
*S* = 0.97	*w* = 1/[σ^2^(*F*_o_^2^) + (0.0012*P*)^2^] where *P* = (*F*_o_^2^ + 2*F*_c_^2^)/3
1685 reflections	(Δ/σ)_max_ < 0.001
124 parameters	Δρ_max_ = 0.28 e Å^−^^3^
0 restraints	Δρ_min_ = −0.35 e Å^−^^3^

### Special details

**Table:**

Geometry. All e.s.d.'s (except the e.s.d. in the dihedral angle between two l.s. planes) are estimated using the full covariance matrix. The cell e.s.d.'s are taken into account individually in the estimation of e.s.d.'s in distances, angles and torsion angles; correlations between e.s.d.'s in cell parameters are only used when they are defined by crystal symmetry. An approximate (isotropic) treatment of cell e.s.d.'s is used for estimating e.s.d.'s involving l.s. planes.
Refinement. Refinement of *F*^2^ against ALL reflections. The weighted *R*-factor *wR* and goodness of fit *S* are based on *F*^2^, conventional *R*-factors *R* are based on *F*, with *F* set to zero for negative *F*^2^. The threshold expression of *F*^2^ > σ(*F*^2^) is used only for calculating *R*-factors(gt) *etc*. and is not relevant to the choice of reflections for refinement. *R*-factors based on *F*^2^ are statistically about twice as large as those based on *F*, and *R*-factors based on ALL data will be even larger.

### Fractional atomic coordinates and isotropic or equivalent isotropic displacement parameters (Å^2^)

**Table:**

	*x*	*y*	*z*	*U*_iso_*/*U*_eq_	
C1	0.6897 (3)	0.3911 (3)	0.06190 (18)	0.0321 (6)	
C2	0.5743 (3)	0.3976 (3)	0.12798 (17)	0.0344 (7)	
C3	0.4233 (3)	0.3110 (3)	0.11860 (18)	0.0383 (7)	
H3A	0.3502	0.3176	0.1631	0.046*	
C4	0.3801 (3)	0.2139 (3)	0.0429 (2)	0.0384 (7)	
C5	0.4853 (3)	0.2045 (3)	−0.02361 (18)	0.0393 (7)	
H5A	0.4546	0.1393	−0.0747	0.047*	
C6	0.6402 (3)	0.2937 (3)	−0.01460 (18)	0.0324 (6)	
Cl1	0.62781 (9)	0.51726 (8)	0.22421 (4)	0.0454 (2)	
Cl2	0.18645 (10)	0.10465 (8)	0.03097 (5)	0.0538 (2)	
Ni1	1.0000	0.5000	0.0000	0.03205 (15)	
O1	0.8324 (2)	0.47509 (19)	0.07451 (12)	0.0393 (5)	
C7	0.7422 (3)	0.2847 (3)	−0.08801 (18)	0.0374 (7)	
H7A	0.7023	0.2171	−0.1369	0.045*	
C8	0.9509 (4)	0.3268 (3)	−0.18214 (19)	0.0465 (8)	
H8A	0.9135	0.2303	−0.2040	0.056*	
H8B	1.0840	0.3302	−0.1659	0.056*	
C9	0.8770 (4)	0.4311 (4)	−0.26300 (19)	0.0654 (10)	
H9A	0.9229	0.4067	−0.3187	0.098*	
H9B	0.9155	0.5265	−0.2419	0.098*	
H9C	0.7453	0.4266	−0.2800	0.098*	
N1	0.8836 (3)	0.3602 (2)	−0.09375 (14)	0.0337 (5)	

### Atomic displacement parameters (Å^2^)

**Table:**

	*U*^11^	*U*^22^	*U*^33^	*U*^12^	*U*^13^	*U*^23^
C1	0.0305 (15)	0.0322 (17)	0.0333 (15)	0.0026 (13)	0.0070 (13)	0.0053 (13)
C2	0.0343 (16)	0.0373 (17)	0.0315 (15)	0.0041 (13)	0.0078 (13)	0.0053 (13)
C3	0.0341 (16)	0.0456 (19)	0.0376 (16)	0.0033 (14)	0.0132 (14)	0.0094 (15)
C4	0.0303 (16)	0.0388 (17)	0.0449 (17)	−0.0045 (14)	0.0068 (14)	0.0099 (15)
C5	0.0387 (16)	0.0399 (18)	0.0376 (17)	−0.0064 (14)	0.0055 (15)	−0.0018 (13)
C6	0.0329 (15)	0.0315 (16)	0.0332 (15)	−0.0005 (13)	0.0084 (13)	0.0012 (13)
Cl1	0.0483 (4)	0.0536 (5)	0.0372 (4)	−0.0018 (4)	0.0162 (4)	−0.0060 (4)
Cl2	0.0394 (4)	0.0634 (6)	0.0583 (5)	−0.0150 (4)	0.0110 (4)	0.0083 (4)
Ni1	0.0334 (3)	0.0326 (3)	0.0319 (3)	−0.0019 (2)	0.0112 (2)	−0.0032 (2)
O1	0.0403 (11)	0.0445 (13)	0.0372 (10)	−0.0116 (10)	0.0173 (9)	−0.0094 (9)
C7	0.0427 (17)	0.0348 (17)	0.0341 (16)	−0.0010 (14)	0.0077 (14)	−0.0038 (13)
C8	0.0481 (18)	0.051 (2)	0.0465 (18)	−0.0117 (15)	0.0238 (15)	−0.0196 (16)
C9	0.055 (2)	0.105 (3)	0.0394 (18)	−0.013 (2)	0.0178 (17)	0.002 (2)
N1	0.0374 (13)	0.0342 (13)	0.0326 (12)	−0.0020 (11)	0.0143 (11)	−0.0027 (11)

### Geometric parameters (Å, °)

**Table:**

C1—O1	1.303 (3)	Ni1—O1^i^	1.8382 (16)
C1—C6	1.393 (3)	Ni1—N1^i^	1.914 (2)
C1—C2	1.419 (3)	Ni1—N1	1.914 (2)
C2—C3	1.372 (3)	C7—N1	1.291 (3)
C2—Cl1	1.731 (3)	C7—H7A	0.9300
C3—C4	1.380 (3)	C8—N1	1.489 (3)
C3—H3A	0.9300	C8—C9	1.502 (4)
C4—C5	1.368 (3)	C8—H8A	0.9700
C4—Cl2	1.749 (3)	C8—H8B	0.9700
C5—C6	1.410 (3)	C9—H9A	0.9600
C5—H5A	0.9300	C9—H9B	0.9600
C6—C7	1.432 (3)	C9—H9C	0.9600
Ni1—O1	1.8382 (16)		
			
O1—C1—C6	123.7 (2)	O1^i^—Ni1—N1	87.10 (8)
O1—C1—C2	119.6 (2)	N1^i^—Ni1—N1	180.0
C6—C1—C2	116.7 (2)	C1—O1—Ni1	130.49 (17)
C3—C2—C1	122.1 (3)	N1—C7—C6	127.1 (3)
C3—C2—Cl1	119.1 (2)	N1—C7—H7A	116.5
C1—C2—Cl1	118.9 (2)	C6—C7—H7A	116.5
C2—C3—C4	119.7 (3)	N1—C8—C9	111.6 (2)
C2—C3—H3A	120.1	N1—C8—H8A	109.3
C4—C3—H3A	120.1	C9—C8—H8A	109.3
C5—C4—C3	120.6 (2)	N1—C8—H8B	109.3
C5—C4—Cl2	119.9 (2)	C9—C8—H8B	109.3
C3—C4—Cl2	119.4 (2)	H8A—C8—H8B	108.0
C4—C5—C6	119.9 (3)	C8—C9—H9A	109.5
C4—C5—H5A	120.1	C8—C9—H9B	109.5
C6—C5—H5A	120.1	H9A—C9—H9B	109.5
C1—C6—C5	121.0 (2)	C8—C9—H9C	109.5
C1—C6—C7	120.8 (2)	H9A—C9—H9C	109.5
C5—C6—C7	118.2 (2)	H9B—C9—H9C	109.5
O1—Ni1—O1^i^	180.00 (13)	C7—N1—C8	112.8 (2)
O1—Ni1—N1^i^	87.10 (8)	C7—N1—Ni1	124.90 (19)
O1^i^—Ni1—N1^i^	92.90 (8)	C8—N1—Ni1	122.30 (17)
O1—Ni1—N1	92.90 (8)		

Symmetry codes: (i) −*x*+2, −*y*+1, −*z*.

## References

[bb1] Aakeröy, C. B., Sinha, A. S., Chopade, P. D. & Desper, J. (2011). *Dalton Trans.* **41**, doi:10.1039/C1DT10911A. 10.1039/c1dt10911a 21845285

[bb2] Bruker (2004). *SMART*, *SAINT* and *SADABS* Bruker AXS Inc., Madison, Wisconsin, USA

[bb3] Cohen, M. D., Schmidt, G. M. J. & Sonntag, F. I. (1964). *J. Chem. Soc.* pp. 2000–2013.

[bb4] Desiraju, G. R. (1989). *Crystal Engineering: The Design of Organic Solids* Amsterdam: Elsevier.

[bb5] Sheldrick, G. M. (2008). *Acta Cryst.* A**64**, 112–122. 10.1107/S0108767307043930 18156677

[bb6] Xiao, Y. & Zhang, M. (2008). *Acta Cryst.* E**64**, m1231. 10.1107/S1600536808026068 PMC2959384 21200991

